# Impact of fermentation on the processing and digestion characteristics of honeysuckle polyphenols by *Lactobacillus acidophilus*

**DOI:** 10.3389/fnut.2025.1570648

**Published:** 2025-05-02

**Authors:** Xiaoyu Dong, Hanhan Shi, Sensen Zhang, Xiaoying Deng, Yongchao Ge, Yongchao Li

**Affiliations:** ^1^School of Agriculture, Henan Institute of Science and Technology, Xinxiang, China; ^2^Henan International Joint Laboratory of Plant Genetic Improvement and Soil Remediation, School of Life Sciences, Henan Institute of Science and Technology, Xinxiang, China

**Keywords:** honeysuckle, *Lactobacillus acidophilus*, fermentation, digestion, processing

## Abstract

The technology and digestive characteristics of honeysuckle beverages fermented by *Lactobacillus acidophilus* were researched, the digestive characteristics were evaluated by simulating the gastrointestinal digestive system *in vitro*. The optimum conditions were as follows: fermentation temperature, 35°C; fermentation time, 19 h; and inoculation amount, 3%. Fermented honeysuckle beverages had greater antioxidant and *α*-glucosidase inhibition capacities than unfermented beverages after digestion. The bioavailabilities of total phenol, total flavonoids and chlorogenic acid in fermented honeysuckle liquid were 29.72, 21.80, and 36.93%, respectively, whereas those in unfermented honeysuckle liquid were 22.03, 17.28, and 25.67%, respectively. The pH of the fermented honeysuckle beverage remained relatively stable during storage at 4°C, with no significant change (*p* > 0.05). The number of viable bacteria decreased from 9.79 lg CFU/mL to 8.31 lg CFU/mL, and the sensory score and color decreased. This study provides a reference basis for the development of honeysuckle products and specific functional probiotic products.

## 1 Introduction

Honeysuckle (*Lonicera japonica* Thunb., LJT) is a common perennial semievergreen vine belonging to the genus *Lonicera* of Caprifoliaceae. Pharmacological studies have shown that honeysuckle has antioxidant, antitumor, liver protection and hypoglycemic properties (1–4). More than 300 compounds, including phenolic acids, flavonoids, saponins, and iridoids, have been isolated and identified from honeysuckle (5, 6). HEP-4, a water-soluble polysaccharide extracted from honeysuckle, has an antioxidant effect on human hepatoma cells (HepG2) (7). Qi et al. (8) prepared a pectin polysaccharide designated WLJP-A0.2b from honeysuckle polysaccharide. Free radical scavenging experiments revealed that WLJP-A0.2b has antioxidant activity through the synergistic effects of different pectin domains. Xu et al. (9) reported that honeysuckle extract can exert its inhibitory effect on *Bacillus cereus* ATCC 14579 by inhibiting ribosome assembly, peptidoglycan biosynthesis and phospholipid biosynthesis, with phenols such as luteolin, quercetin and kaempferol playing major roles. Therefore, exploring the preparation and extraction of bioactive components from honeysuckle and improving the biological characteristics of honeysuckle are highly important for further developing the utilization value of honeysuckle.

When lactic acid bacteria (LAB) ferment, they metabolize nutrients in the culture medium and produce a variety of metabolites, which has a positive impact on human health (10). LAB can enhance the activity of gastric acid and pepsin, improve the digestion and absorption capacity of the gastrointestinal tract, and promote the absorption of nutrients such as calcium and phosphoru, and also decompose cellulose and other substances that are difficult to decompose under normal conditions, making nutrients more easily absorbed by the body (11). LAB lead to the hydrolysis of compounds through enzymatic activities such as glycosidases or decarboxylases, and release phenolics bound to the plant cell wall, improving their bioavailability (12). Abanoz et al. (13) isolated a strain of *Enterococcus faecalis* KT11 from traditional Castiglia cheese. The KT11 bacteriocin can be used to treat multidrug-resistant clinical pathogens alone or in combination with traditional antibiotics. Hasan et al. (14) reported that the bacteriocin of *Lactobacillus casei* can effectively inhibit urogenital tract and intestinal pathogens. Owing to the probiotic effect of lactic acid bacteria, fermented products have become functional products with antioxidant activity, reducing blood lipids and improving immunity.

In this study, *Lactobacillus acidophilus* was used to ferment honeysuckle liquid on the basis of previous experiments, and a response surface optimization test was carried out, with the number of viable bacteria and sensory score as response values. The changes in pH, gastric emptying rate, viable number and survival rate of lactic acid bacteria, content and bioavailability of active substances, antioxidant capacity and *α*-glucosidase inhibition ability of honeysuckle liquid during simulated gastrointestinal digestion were evaluated through the *in vitro* simulated digestive system (DIVRS) model, and changes in the quality of fermented honeysuckle lactic acid bacteria beverages during storage were studied. The results of this study provide a new method for the comprehensive utilization of honeysuckle.

## 2 Materials and methods

### 2.1 Materials strains and reagents

Honeysuckle was obtained from the Honeysuckle Experimental Base of the Henan Institute of Science and Technology, Xinxiang city, Henan Province. *Lactobacillus acidophilus* zrx02 (*L. acidophilus zrx02, LA*, GeneBank NO. MF804413) is a strain preserved by the Food Research Institute of Henan Institute of Science and Technology.

### 2.2 Culture

The strains were inoculated in 10 mL of MRS medium and cultured at 37°C for 12 h. Then, 1 mL of culture medium was added to 100 mL of MRS medium and cultured at 37°C for 12 h. Bacteria were collected by centrifugation (6,000 × g, 10 min, 4°C) and washed twice with an equal volume of sterile saline (0.9% NaCl solution) to obtain a standby bacterial suspension.

### 2.3 Preparation and fermentation of honeysuckle liquid

The honeysuckle was added to distilled water at a ratio of 3 g/100 g, and sucrose was added as carbon and energy source at a ratio of 7 g/100 g. The mixture was then pasteurized at 60°C for 30 min before fermentation. After cooling to room temperature, the bacterial suspension was added to the sterilized honeysuckle mixture at a ratio of 3 mL/100 mL and fermented at 37°C for 24 h. The honeysuckle liquid fermented without lactic acid bacteria under the same conditions was used as a control.

### 2.4 Response surface test

On the basis of the single factor test results, three single factors, fermentation temperature (A), fermentation time (B) and inoculation amount (C), were selected, and three test levels were designed for each factor. The Box–Behnken response surface optimization design was carried out with the number of viable bacteria and the sensory score as indicators. See Table 1 for factors and levels. The experiment was repeated three times under the optimization conditions, and the validation experiment was carried out with defined parameters by comparing the significance between the actual values and the theoretical values.

**Table 1:** Box–Behnken design of fermentation temperature, fermentation time, and inoculation amount of honeysuckle fermentation beverage process.

Level	Factors		
	A: Fermentation temperature (°C)	B: Fermentation time (h)	C: Inoculation amount (%)
−1	33	12	2.0%
0	35	18	3.0%
1	37	24	4.0%

### 2.5 Determination of viable bacteria

The number of viable bacteria and the physical and chemical properties of the honeysuckle liquid were determined according to the methods of Wang et al. (15). The number of viable LAB was measured on MRS agar plates. The samples were continuously diluted to 10^−5^ ~ 10^−8^ with sterile normal saline. The 100 μL diluted samples were coated on MRS agar plates and cultured in a 37°C incubator for 36 ~ 48 h. Then, the number of cells containing 30 ~ 300 colonies on the plate was measured and recorded as log CFU/mL.

### 2.6 Sensory evaluation

The sensory indices, such as color, taste, smell and tissue state, of the honeysuckle beverages were comprehensively evaluated by 10 evaluators (5 males and 5 females) with basic knowledge of food sensory evaluation. The scores were color (25 points), taste (25 points), smell (25 points) and tissue state (25 points), with a total possible score of 100 points. See Table 2 for the scoring criteria.

**Table 2:** Sensory scoring criteria for honeysuckle fermented beverage.

Sensory	Evaluation criteria	Score
Color (25)	Honeysuckle liquid color coordination, bright and shiny color	18 ~ 25
	The color of honeysuckle liquid is basically coordinated, and the color is general	9 ~ 17
	Honeysuckle liquid color is not harmonious, color is not uniform	0 ~ 8
Taste (25)	The taste is pure, fresh and refreshing	18 ~ 25
	Taste general, slightly bitter taste	9 ~ 17
	Taste slightly worse, there is a great bitterness	0 ~ 8
Smell (25)	The smell is coordinated and has a rich flavor of honeysuckle	18 ~ 25
	The smell is basically coordinated, and the flavor of honeysuckle is slightly light	9 ~ 17
	Odor is not harmonious, honeysuckle flavor is not good	0 ~ 8
Tissue state (25)	The state is uniform, no impurities, no precipitation appears	18 ~ 25
	The state is uniform, with slight precipitation	9 ~ 17
	The state is not uniform, and there is obvious precipitation	0 ~ 8

### 2.7 DIVRS model

The disposition method for digestive juices, such as saliva, gastric juice and intestinal juice, was based on the method of Zhang et al. (16). CaCl_2_ solution: A weight of 4.41 g of CaCl_2_(H_2_O)_2_ was dissolved in an appropriate amount of double-distilled water. Then, make up the volume to 100 mL in a volumetric flask. The concentration of the prepared solution was 0.3 mol/L. Simulated Gastric Fluid (SGF) (200 mL): A volume of 160 mL SGF electrolyte stock solution + 100 μL of CaCl₂ + 38.9 mL of deionized water + 1.2308 g of pepsin. The activity of pepsin was 3,000 U/mg. Adjust the pH to 1.6 during the dynamic *in vitro* digestion process. Simulated Intestinal Fluid (SIF) (200 mL): A volume of 160 mL of SIF electrolyte stock solution + 400 μL of CaCl₂ + 38.9 mL of deionized water + 1.246 g of pancreatin + 136.8 mg of porcine bile salts. The activity of pancreatin was 130 U/mg, and adjust the pH to 7. The DIVRS model was activated, and the incubator temperature was set to 37°C for 30 min of preheating. The prepared gastric juice and intestinal juice were separately filled into 25 - mL special syringes. Air was purged, and the silicone digestion pipes were connected. The pylorus, gastric pressure plate, stomach roller, and intestinal roller buttons on the control panel were manually operated to adjust the movement frequency, ensuring that the empty pump and the injection pump were in appropriate positions. A 6 - mL volume of the sample to be tested was taken, to which 1 mL of saliva simulation solution was added. The mixture was thoroughly homogenized and then incubated in a 37°C water bath for 5 min to simulate oral digestion of the sample. Subsequently, the sample was transferred into the bionic stomach (maintained at 37°C). The compression device was configured to generate 3 compressions per minute, and the amplitude of the angle plate was set at 2.6 mm. These settings were determined to mimic the contraction movements occurring *in vivo*. After digestion, the residual samples from the stomach and intestine were collected and stored for later use after enzyme inactivation.

The equipment operating parameters were as follows: temperature, 37°C; simulated gastric juice injection speed, 25 μL/min; simulated intestinal fluid injection speed, 30 μL/min; emptying speed, 100 μL/min; gastric compression frequency, 0.1 × g; gastric rolling frequency, 0.36 × g; and intestinal rolling frequency, 0.98 × g.

### 2.8 pH determination

After digestion in the DIVRS model for 30 min, 60 min, 90 min, 120 min, and 150 min, the digestive products in the stomach and intestine were removed, and the pH values of the samples were measured directly with a calibrated pH meter.

### 2.9 Determination of the gastric emptying rate

After digestion with DIVRS for 30 min, 60 min, 90 min, 120 min, and 150 min, the volume of chyme retained in the stomach was measured immediately. The calculation of the gastric emptying rate was based on the method of Feng et al. (17) and was performed according to Equation 1:


(1) $$\mathrm{Gastric emptying rate}\;\left(\%\right)=\frac{A_{0}-A_{1}}{A_{0}}\times 100$$

A_0_ represents the initial volume of the substances (including samples and gastric juice) entering the stomach at a given time point; A_1_ represents the volume of chyme retained in the stomach at a given time point.

### 2.10 Determination of viable bacteria after gastrointestinal digestion

In accordance with the methods of Wu et al. (18), the gastric and intestinal chyme swirls digested continuously for 30, 60, 90, 120 and 150 min were mixed evenly and then diluted in a gradient of sterile normal saline. Colony counting was performed at 37°C for 48 h. Colony counts are expressed as N0, N30, N60, N90, N120 and N150 CFU/mL. The number of viable bacteria during digestion was compared with that before digestion, and the survival rates at 30, 60, 90, 120, and 150 min after gastrointestinal digestion were obtained. The survival rates of lactic acid bacteria in the stomach and intestine at different digestion times were calculated according to Equations 2, 3 (18), respectively:


(2) $$GT\;\min\;\left(\%\right)=\frac{N_{T,G}}{N_{0}}\frac{C_{T,G}V_{T,G}}{C_{0}V_{0}}\times 100$$

(3) $$\mathrm{IT}\;\min\;\left(\%\right)=\frac{N_{T,I}}{N_{0}}\frac{C_{T,I}V_{T,I}}{C_{0}V_{0}}\times 100$$

where T represents the digestion time, min; GT min represents the survival rate of *Lactobacillus* in the stomach after T min of digestion, %; IT min represents the survival rate of lactic acid bacteria in the intestinal tract after T min of digestion,%; N_T_,_G_ represent the number of viable lactic acid bacteria in the stomach after T min of digestion; N_T_,_I_ represent the number of viable lactic acid bacteria in the intestinal tract after T min of digestion; N_0_ represents the number of viable bacteria in the initial lactic acid bacteria of the sample; C_T_,_G_ represent the concentration of lactic acid bacteria in the stomach after T min of digestion, CFU/mL; V_T_,_G_ represent the volume, mL, of the sample in the stomach after T min of digestion; C_T_,_I_ represent the concentration of lactic acid bacteria in the intestinal tract after T min of digestion, CFU/mL; and V_T_,_I_ represent the volume, mL, of the sample in the intestine after T min of digestion.

### 2.11 Determination of the content of active ingredients

#### 2.11.1 Total phenol content

The Folin–Ciocalteu method was used (19). A volume of 1 mL of the diluted sample was reacted with 5 mL of 10% (w/v) Folin–Ciocalteu solution for 5 min and then mixed with 4 mL of Na_2_CO_3_ solution (75 g/L) for 40 min. The absorbance was measured at 765 nm. The results are expressed as gallic acid equivalents (mg GAE/100 mL).

#### 2.11.2 Total flavonoid content

AlCl_3_ colorimetry was used (20). A volume of 4 mL of the diluted sample was mixed with 0.5 mL of NaNO_2_ solution (50 g/L) for 5 min. After 1 mL of AlCl_3_ solution (100 g/L) was added, the reaction was allowed to continue for 5 min; 2 mL of NaOH solution (2 mol/L) was then added, and the reaction was allowed to continue for 10 min, after which the absorbance was measured at 510 nm. The results are expressed as rutin equivalents (mg RE/100 mL).

#### 2.11.3 Chlorogenic acid content

High-performance liquid chromatography was used (21). A 1 mL sample was centrifuged at 4°C for 15 min at 7000 × g. The supernatant was filtered with a 0.22 μm filter membrane and injected into a high-performance liquid chromatography system (Waters Inc., USA) on a TC-C18 column of 250 mm × 4.6 mm, 5 μm (Agilent, USA). The temperature was 30°C. The detection wavelength was 320 nm. The injection volume was 20 μL. The volume flow rate was 0.6 mL/min. The mobile phase was 0.1% (v/v) formic acid aqueous solution (A) and methanol solution (B). The gradient elution conditions were as follows: 0 ~ 10 min, 5% ~ 30% B; 10 ~ 25 min, 30% ~ 70% B; 25 ~ 35 min, 50% ~ 70% B; and 35 ~ 40 min, 70% ~ 5% B. The results were expressed as chlorogenic acid equivalents (mg CGA/100 mL).

### 2.12 Determination of antioxidant capacity

#### 2.12.1 Free radical scavenging activity of DPPH

The method of Qi et al. (22) was used, with some modifications. A 2 mL sample diluted 5 times or distilled water (control) was added to 4 mL of DPPH solution (0.2 mmol/L) for 30 min, and the absorbance was measured at 517 nm. DPPH radical scavenging activity was calculated according to Equation 4:


(4) $$\begin{array}{l} \mathrm{DPPH free radical}\;\\ \;\mathrm{scavenging rate}\;\left(\%\right)=\frac{A_{\mathrm{control}}-A_{\mathrm{sample}}}{A_{\mathrm{control}}}\times 100\; \end{array}$$

A_control_ represents the absorbance of distilled water; A_sample_ represents the absorbance of the sample.

#### 2.12.2 Hydroxyl radical scavenging activity

The method of Zhang et al. (23) was used with some modifications. One milliliter of 2-fold diluted sample or distilled water (control) was added to 1 mL of 1,10-phenanthroline solution (0.75 mmol/L) and 2 mL of sodium phosphate buffer (PBS, pH 7.4). After mixing, 1 mL of FeSO_4_ solution (0.75 mmol/L) and 1 mL of H_2_O_2_ solution (0.12%, v/v) were added. The reaction was carried out at 37°C for 30 min, and the absorbance was measured at 536 nm. The hydroxyl radical scavenging activity was calculated according to Equation 5:


(5) $$OH\;\mathrm{free radical scavenging rate}\;\left(\%\right)=\frac{A_{\mathrm{sample}}-A_{\mathrm{blank}}}{A_{\mathrm{control}}-A_{\mathrm{blank}}}\times 100$$

A_control_ represents the absorbance of a sample prepared with distilled water; A_sample_ represents the absorbance of a sample; and A_blank_ represents the absorbance of a sample that contained distilled water but not H_2_O_2_.

#### 2.12.3 Radical scavenging activity of superoxide anions

The pyrogallol autoxidation method was used for determination (24). One milliliter of sample solution or distilled water (control) was added to 2.4 mL of distilled water, and 4.5 mL of Tris–HCl buffer (0.1 mmol/L, pH = 8.2) was added, followed by mixing and adding 0.1 mL of pyrogallol solution (30 mmol/L), after which the absorbance was measured at 325 nm. The superoxide anion radical scavenging activity was calculated according to Equation 6:


(6) $$O_{2}^{-}\mathrm{free radical scavenging rate}\;\left(\%\right)=\frac{A_{\mathrm{control}}-A_{\mathrm{sample}}}{A_{\mathrm{control}}}\times 100$$

The A_control_ represents the absorbance of a sample prepared with distilled water; the A_sample_ represents the absorbance of a sample.

#### 2.12.4 Determination of reducing ability

The method of Martínez-Las et al. (25) was used, and some modifications were made. A 0.5 mL sample was added to 2.5 mL of phosphate buffer (0.2 mol/L, pH 6.6) and 2.5 mL of 1% (w/v) potassium ferricyanide solution, and the mixture was incubated for 20 min at 50°C. Then, 2.5 mL of 10% (v/v) trichloroacetic acid solution was added, the mixture was centrifuged for 10 min (4,000 r/min), 2.5 mL of the supernatant was mixed with 2.5 mL of distilled water and 0.5 mL of 0.1% (w/v) ferric chloride solution, and the absorbance was measured at 700 nm. The greater the absorbance was, the greater the reducing ability.

### 2.13 Inhibition of *α*-glucosidase

The method of Zhang et al. (23) was used with some modifications. A volume of 100 μL of sample or distilled water (control) was added to 100 μL of α-glucosidase solution (0.5 U/mL) and reacted at 37°C for 10 min. Then, 100 μL of pNP-G solution (5 mmol/L) was added for 20 min, and the absorbance was recorded with a microplate reader at 405 nm. The α-glucosidase inhibitory capacity was calculated according to Equation 7:


(7) $$\alpha -\mathrm{glucosidase inhibitory ability}\;\left(\%\right)=\frac{A_{\mathrm{control}}-A_{\mathrm{sample}}}{A_{\mathrm{control}}}\times 100$$

The A_control_ represents the absorbance of a sample with distilled water, and the A_sample_ represents the absorbance of a sample.

### 2.14 Changes in product quality during storage

The mixed honeysuckle lactic acid bacteria beverage was stored at 4°C, and the changes in pH, number of viable lactic acid bacteria, color characteristics and sensory scores were studied at 0, 3, 6, 9, 12 and 15 days. The pH was measured according to the method outlined in section 2.8, the number of viable bacteria was measured according to the method outlined in section 2.5, and the sensory score was measured according to the method outlined in section 2.6.

The color characteristics were determined as follows: a Hunter colorimeter was used to determine the color characteristics of the honeysuckle liquid (L*, a*, and b*), where L* indicated brightness (the higher the L value was, the more white the color was), A* indicated a red–green color (a positive value indicated that the color was biased toward red, and a negative value indicated that the color was biased toward green), B* denoted a yellowish–blue color (a positive value denoted a color bias toward yellow, and a negative value denoted a color bias toward blue). ΔE indicated the total color difference of the fermented sample compared with that of the control (unfermented sample). The larger the ΔE value was, the more obvious the color difference was. ΔE was calculated according to Equation 8:


(8) $$\Delta E=\sqrt{{\left(L_{0}*-L*\right)}^{2}+{\left(a_{0}*-a*\right)}^{2}+{\left(b_{0}*-b*\right)}^{2}}$$

L_0_*, A_0_* and B_0_* represent the values of L, A and B, respectively, in the control group, and L*, A* and B* represent the values of L, A and B, respectively, in the fermentation group.

### 2.15 Data analysis

All the experiments were repeated 3 times. The results are expressed as the means ± standard deviations. SPSS 20.0 was used for one-way analysis of variance (ANOVA). *p* < 0.05 was considered statistically significant.

## 3 Results

### 3.1 Response surface optimization test and variance analysis

According to the results of the single factor test, three single factors, namely, fermentation temperature (A), fermentation time (B) and inoculation amount (C), were selected for the response surface optimization test. The results in Table 3 were analyzed by Design Expert 8.0.6 software, and the regression equations of the viable bacteria count (Y1) and sensory score (Y2) with fermentation temperature (A), fermentation time (B) and inoculum (C) were obtained as follows:


$$\begin{array}{l} Y1=9.91+0.07A+0.15B-0.06C+0.08\mathrm{AB}-0.01\;\mathrm{AC}\\ \;-0.02\;BC-0.44A2-0.38B2-0.42C2 \end{array}$$

$$\begin{array}{l} Y2=83.58+1.79A+1.39B-0.83C+0.65\mathrm{AB}\\ \;-0.58\;\mathrm{AC}-0.23\;BC-4.14A2-5.04B2-3.22C2 \end{array}$$

**Table 3:** Design and results of response surface of honeysuckle fermented beverage.

Test number	A Fermentation temperature (°C)	B Fermentation time (h)	C Inoculation amount (%)	Y_1_ Viable cell counts (lg CFU/mL)	Y_2_ Sensory score
1	33	24	3	9.08	73.3
2	35	18	3	9.89	82.8
3	37	12	3	8.93	74.2
4	35	18	3	9.92	83.9
5	33	18	4	8.96	74.3
6	37	18	4	9.01	76.3
7	35	12	4	8.95	73.6
8	33	18	2	9.06	75.0
9	35	18	3	9.94	83.6
10	35	18	3	9.91	83.5
11	37	24	3	9.44	78.6
12	35	24	4	9.15	75.6
13	33	12	3	8.89	71.5
14	35	24	2	9.31	77.5
15	35	12	2	9.04	74.6
16	35	18	3	9.87	84.1
17	37	18	2	9.16	79.3

The results of the variance and significance analysis of the regression model of the number of viable bacteria are shown in Table 4. The regression model between the number of viable bacteria and various variables in the model was *p* < 0.0001, which represented a very significant level, whereas the misfit term of the equation was *p* = 0.0501 > 0.05, which was not significant, indicating that the degree of fit of this model was good. The regression coefficient R^2^ = 0. 9,936, and the correction coefficient R^2^Adj = 0.9853 of the model showed that the model fit well with the experimental results and that the error was small. The model had a high degree of fit with the actual results, which better reflected the influence of the three factors on the number of viable bacteria in the fermented honeysuckle liquid. Therefore, this model can be used to analyze and predict the fermentation conditions of honeysuckle liquid. In this model, the primary terms A (fermentation temperature), B (fermentation time), and C (inoculation amount) and the secondary terms A2, B2, and C2 all had significant effects on the number of viable bacteria in the fermented honeysuckle liquid (*p* < 0.01), the interactive term AB had a significant effect on the number of viable bacteria in the fermented honeysuckle liquid (*p* < 0.05), and the interactive terms AC and BC had no significant effect on the number of viable bacteria in the fermented honeysuckle liquid (*p* > 0.05). The *F* value revealed that the influence of each factor on the number of viable bacteria in the fermented honeysuckle liquid was as follows: B (fermentation time) > A (fermentation temperature) > C (inoculation amount).

**Table 4:** Variance analysis of regression model for the number of honeysuckle fermented beverage viable bacteria.

Source	Sum of squares	Degree of freedom	Mean square	*F-*value	*p*-value	Significance
Model	2.67	9	0.30	119.93	< 0.0001	**
A-Fermentation temperature	0.04	1	0.04	15.26	0.0059	**
B-Fermentation time	0.17	1	0.17	69.06	< 0.0001	**
C-Inoculation amount	0.03	1	0.03	12.61	0.0093	**
AB	0.03	1	0.03	10.33	0.0148	*
AC	0.001	1	0.001	0.25	0.6309	
BC	0.001	1	0.001	0.49	0.5047	
A^2^	0.83	1	0.83	333.48	< 0.0001	**
B^2^	0.60	1	0.60	242.80	< 0.0001	**
C^2^	0.73	1	0.73	293.36	< 0.0001	**
Residual	0.02	7	0.002			
Misfit	0.01	3	0.005	6.59	0.0501	
Pure Error	0.003	4	0.001			
Total	2.69	16				

R^2^ = 0.9936; R^2^_Adj_ = 0.9853.*Significant difference, *p* < 0.05; the difference of ** was extremely significant, *p* < 0.01.

The results of the variance and significance analysis of the regression model of the number of viable bacteria are shown in Table 5. The regression model between the sensory score and various variables in the model was *p* < 0.0001, which represented a very significant level, whereas the misfit term of the equation was *p* = 0.5225 > 0.05, which was not significant, indicating that the degree of fit of this model was good. The regression coefficient R^2^ = 0.9945 and the correction coefficient R^2^Adj = 0.9875 of the model showed that the model fit well with the experimental results and that the error was small. The model had a high degree of fit with the actual results, which can better reflect the influence of the three factors on the sensory score of fermented honeysuckle liquid. Therefore, this model can be used to analyze and predict the fermentation conditions of honeysuckle liquid. In the model, the effects of primary terms A (fermentation temperature), B (fermentation time), C (inoculation amount), and secondary terms A2, B2, and C2 on the sensory score of the fermented honeysuckle beverages reached a very significant level (*p* < 0.01), the effects of interactive terms AB and AC on the sensory score of the fermented honeysuckle beverages reached a significant level (*p* < 0.05), and the effects of interactive terms BC on the sensory score of the fermented honeysuckle beverages were not significant (*p* > 0.05). The effects of various factors on the sensory score of the fermented honeysuckle beverages according to the *F* value were as follows: A (fermentation temperature) > B (fermentation time) > C (inoculation amount).

**Table 5:** Variance analysis of regression model for sensory score of honeysuckle fermented beverage.

Source	Sum of squares	Degree of freedom	Mean square	*F-*value	*P*-value	Significance
Model	297.28	9	33.03	140.94	< 0.0001	**
A-Fermentation temperature	25.56	1	25.56	109.07	< 0.0001	**
B-Fermentation time	15.40	1	15.40	65.72	< 0.0001	**
C-Inoculation amount	5.45	1	5.45	23.23	0.0019	**
AB	1.69	1	1.69	7.21	0.0313	*
AC	1.32	1	1.32	5.64	0.0492	*
BC	0.20	1	0.20	0.86	0.3835	
A^2^	72.17	1	72.17	307.93	< 0.0001	**
B^2^	106.95	1	106.95	456.37	< 0.0001	**
C^2^	43.52	1	43.52	185.70	< 0.0001	**
Residual	1.64	7	0.23			
Misfit	0.65	3	0.22	0.88	0.5225	
Pure Error	0.99	4	0.25			
Total	298.92	16				

R^2^ = 0.9945; R^2^_Adj_ = 0.9875. *Significant difference, *p* < 0.05; the difference of ** was extremely significant, *p* < 0.01.

**Figure 1:**
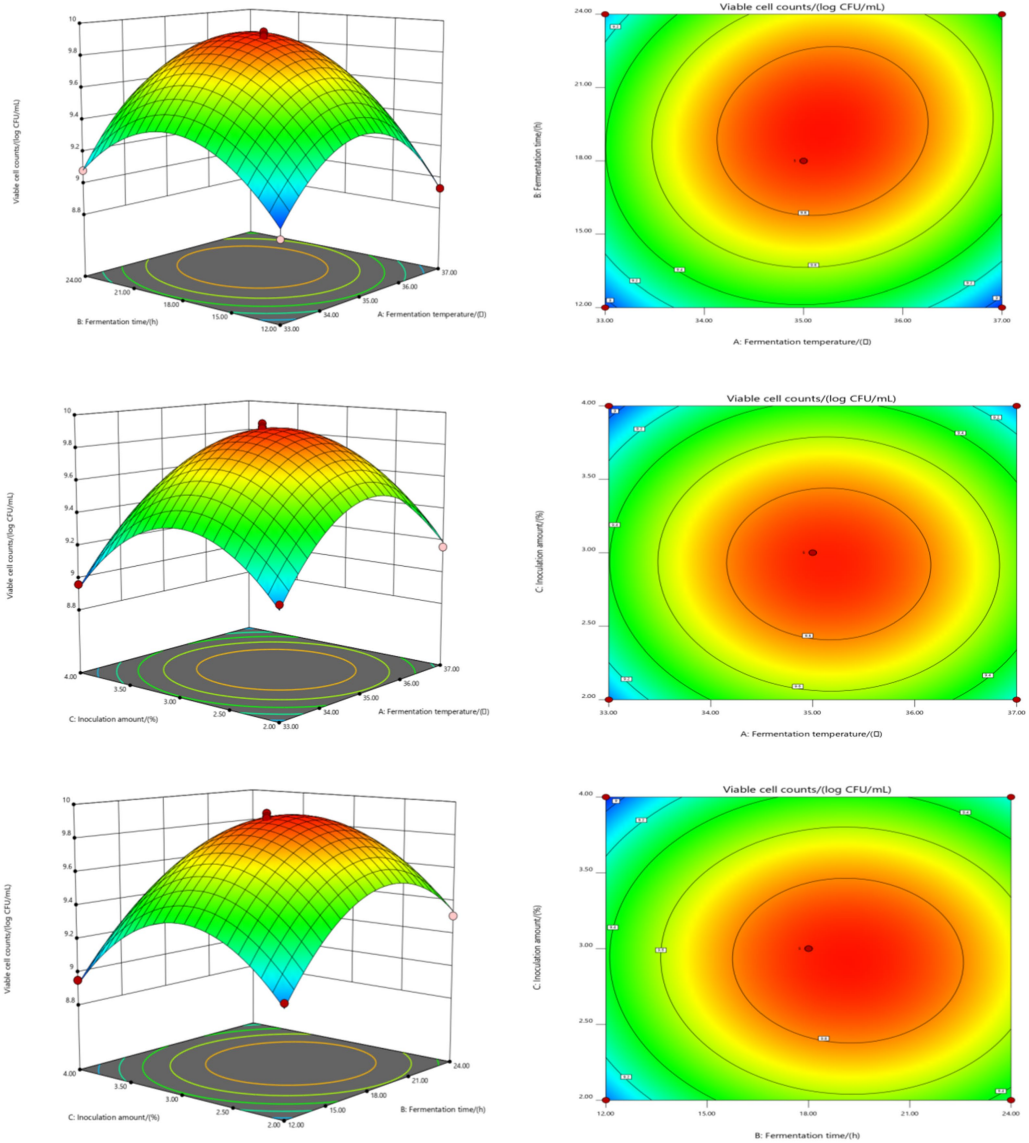
Effect of interaction among fermentation temperature, fermentation time, and inoculation amount on the number of viable bacteria in fermented honeysuckle extract.

Figure 1 shows the influence of interactions among factors on the number of viable bacteria in the fermented honeysuckle liquid. For the AB interaction surface, the slope of the response surface was steep, and the contour lines were dense (Figure 1), which revealed that the interaction between A and B had a significant influence on the number of viable bacteria in the fermented honeysuckle liquid (*p* < 0.05). The fermentation temperature and fermentation time strongly influenced the number of viable bacteria in the fermented honeysuckle liquid. When the fermentation temperature was constant, with increasing fermentation time, the number of viable bacteria in the fermented honeysuckle liquid first increased but then decreased. When the fermentation time was fixed, with increasing fermentation temperature, the number of viable bacteria first increased but then decreased, which was consistent with the results of the single-factor test. However, for the AC and BC interaction surfaces, the interactions between fermentation temperature and inoculation amount and between fermentation time and inoculation amount were relatively weak, and there was no significant effect on the number of viable bacteria in the fermented honeysuckle liquid (*p* > 0.05). The above results were consistent with the results of the variance analysis in Table 4.

**Figure 2:**
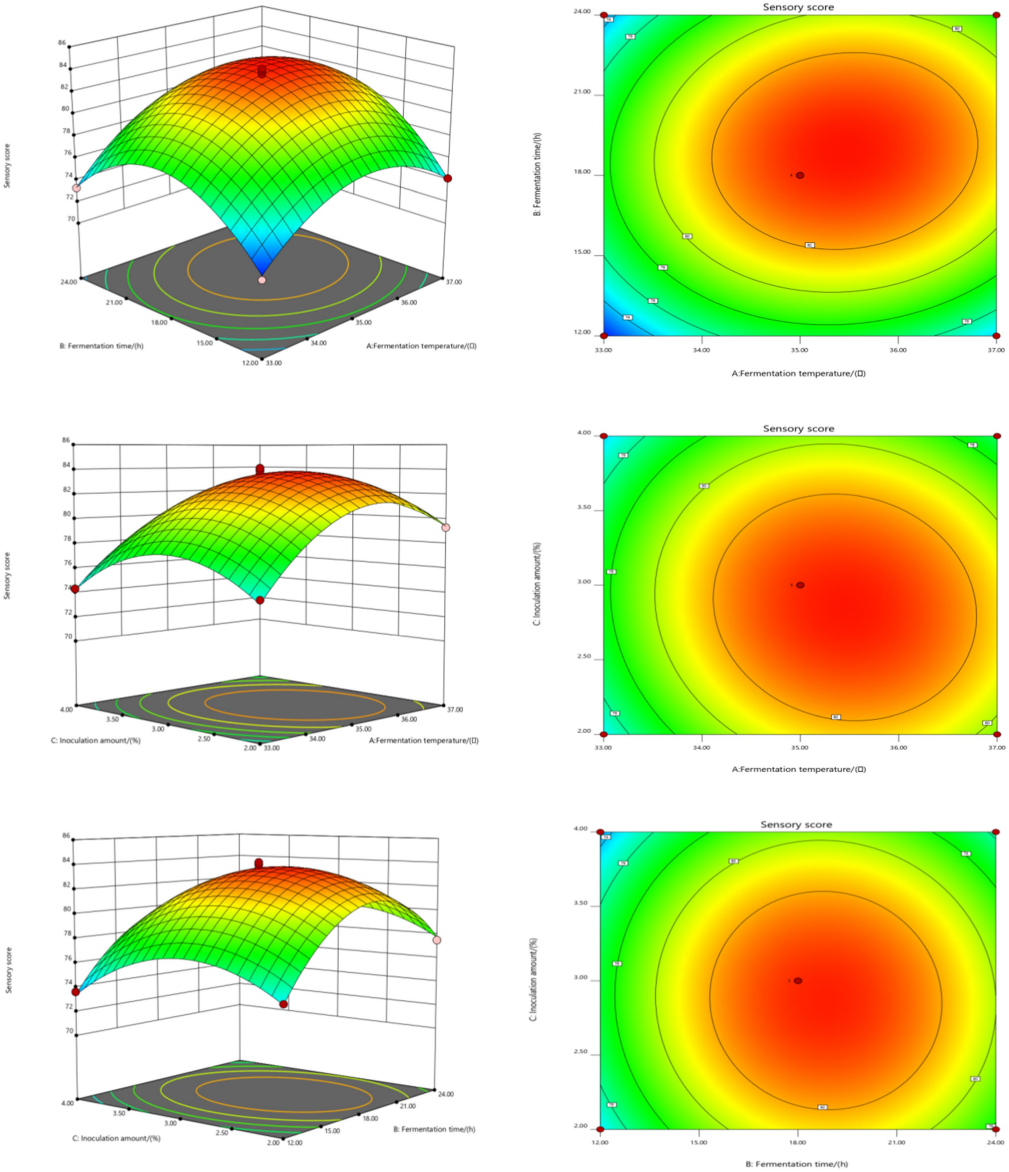
Effect of interaction among fermentation temperature, fermentation time, and inoculation amount on sensory score of fermented honeysuckle extract. Different capital letters indicate significant differences between different digestive sites at the same digestion time (*p* < 0.01); different lowercase letters indicated that there was a significant difference in digestion time between the same digestive site (*p* < 0.05).

The influence of interactions among factors on the sensory score of fermented honeysuckle beverages is shown in Figure 2. With increasing fermentation temperature, fermentation time and inoculation amount, the sensory score of the fermented honeysuckle liquid first increased but then decreased, which was consistent with the results of the single-factor experiments. Among the interaction surfaces of AB and AC, the slope of the response surface was steep, and the contour distribution was dense, which indicated that the interactions between A and B and between A and C had a significant influence on the sensory score of the fermented honeysuckle liquid (*p* < 0.05), fermentation temperature and fermentation time and that the fermentation temperature and inoculation amount had a great influence on the sensory score of the fermented honeysuckle liquid. When the fermentation temperature was constant, with increasing fermentation time or inoculation amount, the sensory score of the fermented honeysuckle liquid first increased but then decreased. When the fermentation time or inoculation amount was fixed, the sensory score first increased but then decreased with increasing fermentation temperature. However, the BC interaction surface data revealed that the interaction between fermentation time and inoculation amount had no significant effect on the sensory score of the fermented honeysuckle liquid (*p* > 0.05), which was consistent with the results of the variance analysis shown in Table 5.

According to the results of the response surface test, the optimum fermentation conditions of the honeysuckle liquid were as follows: fermentation temperature, 35.32°C; fermentation time, 19.08 h; and inoculation amount, 2.89%. Under these conditions, the number of viable bacteria and the sensory score of the fermented honeysuckle liquid were 9.92 lg (CFU/mL) and 83.93 points, respectively. To verify the accuracy of the response surface test results, the above fermentation conditions were used for the test. Considering the convenience of practical operation, the optimum fermentation conditions were adjusted to a fermentation temperature of 35°C, fermentation time of 19 h and inoculum amount of 3%. Three parallel validation experiments were carried out under the adjusted fermentation conditions. The actual viable bacteria number and sensory score were 9.81 ± 0.12 lg (CFU/mL) and 83.30 ± 0.67, respectively, which were close to the theoretical predicted values of the model, indicating that the model was in good agreement with the actual results. Therefore, this model can be used to predict the number of viable bacteria and sensory score in the actual fermentation process of honeysuckle liquid.

### 3.2 Changes in the pH of fermented honeysuckle liquid during gastrointestinal digestion

**Figure 3:**
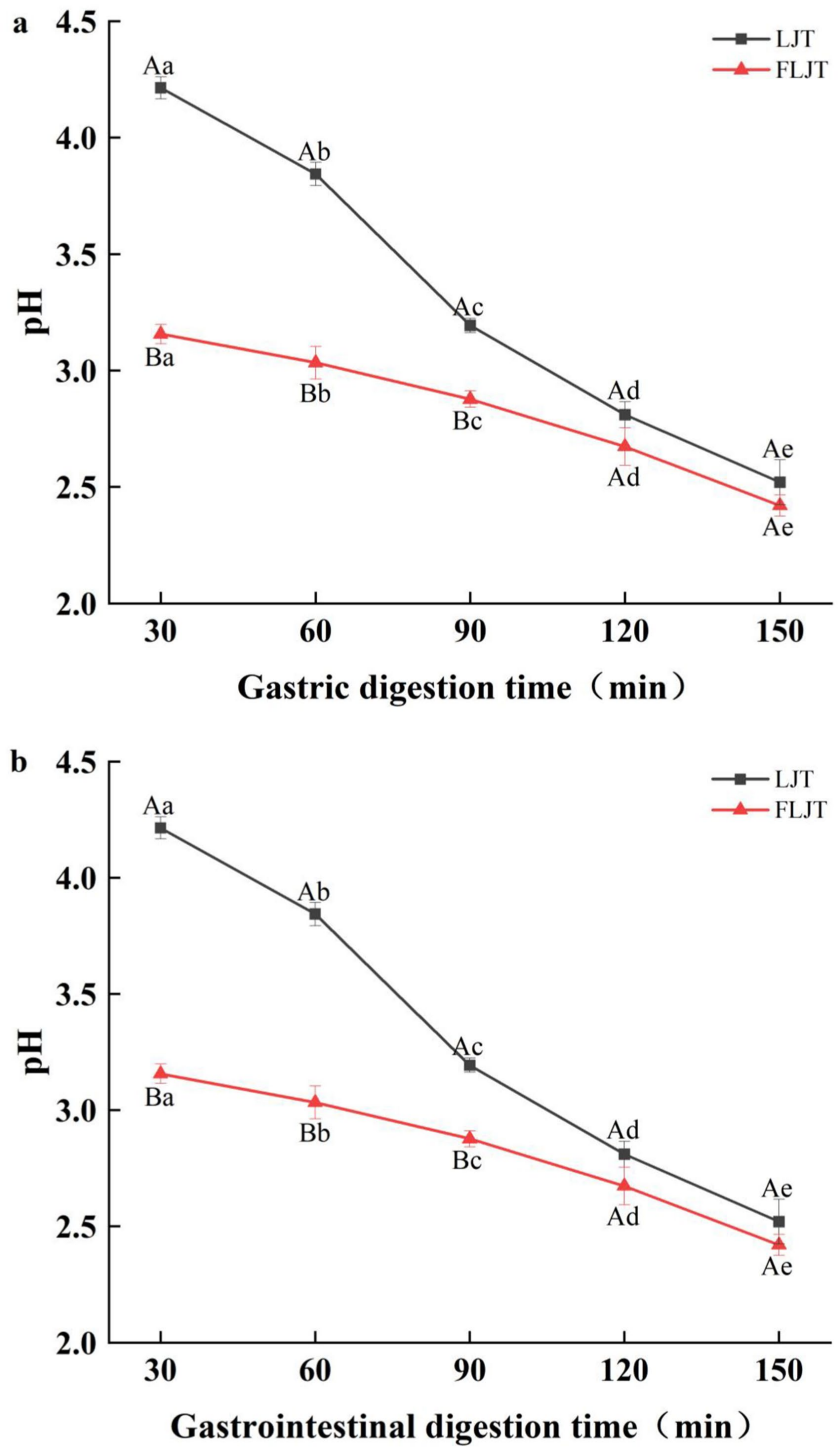
Effects of different digestion time of DIVRS model on the pH of fermented honeysuckle extract: **(a)** gastric digestion phase; **(b)** gastrointestinal digestion phase. Different capital letters in the broken line indicate significant differences between different digestive sites at the same digestion time (*p* < 0.01); different lowercase letters in the broken line indicated that there was a significant difference in digestion time between the same digestive site (*p* < 0.05).

During the gastric digestion stage of in the DIVRS digestion model, the pH of the fermented honeysuckle solution and unfermented honeysuckle solution decreased significantly (*p* < 0.05), and the pH of fermented honeysuckle solution was always lower than that of unfermented honeysuckle solution (Figure 3). The low pH of the fermented honeysuckle liquid was due to the accumulation of acidic substances such as organic acids produced by lactic acid bacteria during fermentation. Owing to the continuous secretion of simulated gastric juice (pH = 1.6) during digestion, the pH of the unfermented honeysuckle solution changed greatly in the early stage of gastric digestion (before 90 min), whereas that of the fermented honeysuckle solution changed little. The pH difference between the fermented and unfermented honeysuckle solutions decreased at the later stage of gastric digestion (after 90 min), especially at 120 ~ 150 min, and the pH values were very close. These findings indicated that the pH of fermented honeysuckle liquid was relatively stable during gastric digestion. During the gastrointestinal digestion stage in the DIVRS digestion model, the pH of the fermented and unfermented honeysuckle liquids increased, which was caused by the continuous secretion of simulated intestinal liquid (pH = 7) during gastrointestinal digestion, but the pH of the fermented and unfermented honeysuckle liquids changed little throughout the whole gastrointestinal digestion process, which indicated that the pH of the fermented and unfermented honeysuckle liquids were relatively stable during gastrointestinal digestion.

### 3.3 Determination of the gastric emptying rate of fermented honeysuckle liquid during digestion

**Figure 4:**
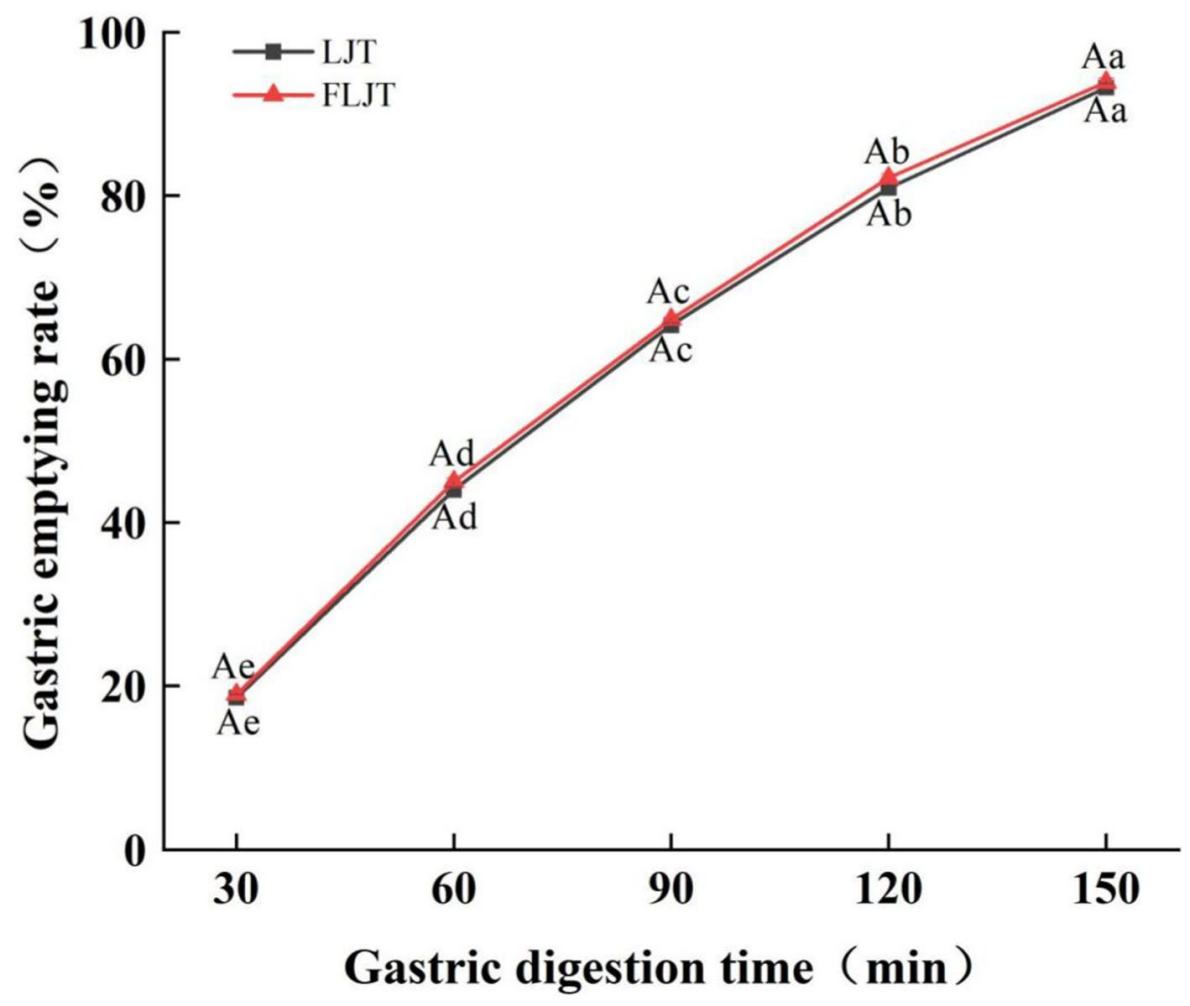
Effects of different digestion time of DIVRS model on gastric emptying rate of fermented honeysuckle extract. LJT is honeysuckle liquid, FLJT is fermented honeysuckle liquid.

As shown in Figure 4, the fermented honeysuckle liquid and unfermented honeysuckle liquid rapidly emptied after ingestion in the gastric model, and the emptying rate of the honeysuckle liquid gradually increased with increasing digestion time. Under the action of peristalsis and contraction in the gastric model, the volume of the fermented honeysuckle liquid and unfermented honeysuckle liquid that emptied from the stomach through the pylorus into the duodenum reached more than 64% at 90 min, which may be one of the reasons for the rapid decrease in the pH of the unfermented honeysuckle liquid at 90 min before gastric digestion, and there was no significant difference in the gastric emptying rate between the fermented honeysuckle liquid and the unfermented honeysuckle liquid at the same digestion time (*p* > 0.05). Therefore, fermentation did not significantly affect the digestion or emptying efficiency of the honeysuckle liquid in the stomach.

### 3.4 Effects of different digestion times on the number of viable bacteria and survival rate in fermented honeysuckle liquid

The effects of *in vitro* digestion on the number and survival rate of lactic acid bacteria in fermented honeysuckle liquid are shown in Table 6. The number of viable *Lactobacillus acidophilus* bacteria in the fermented honeysuckle liquid decreased gradually during digestion, and at the end of digestion, the number of viable *Lactobacillus acidophilus* bacteria in the stomach and intestine decreased significantly compared with that before digestion (*p* < 0.05). The number of viable *Lactobacillus acidophilus* bacteria in the stomach decreased by one order of magnitude, and that in the intestine decreased by two orders of magnitude, from 9.81 lg CFU/mL before digestion to 8.69 lg (CFU/mL) and 7.72 lg (CFU/mL), respectively. The survival rates of *Lactobacillus acidophilus* in the stomach and intestine were 3.61 and 1.71%, respectively. The reason for the decrease in the number of viable bacteria may be that the concentration of the fermentation substrate decreased continuously with the addition of simulated gastric juice and digestive enzymes, and then *Lactobacillus acidophilus* gradually decreased due to the influence of the low pH environment of the gastric juice, which led to the decrease in lactic acid bacteria upon continuous digestion. The number of viable bacteria in the fermented honeysuckle liquid during simulated digestion was greater than 7 lg (CFU/mL), which is in accordance with the number of probiotic bacteria needed to promote human health in the intestinal tract.

**Table 6:** Effects of different digestion time on the number of viable bacteria and survival rate of fermented honeysuckle extract.

Digestion time (min)	Viable cell counts [lg (CFU/mL)]		Survival rate (%)	
	Stomach	Intestine	Stomach	Intestine
30	9.63 ± 0.05^Aa^	9.52 ± 0.03^Ba^	64.26 ± 0.82^Aa^	19.53 ± 0.78^Ba^
60	9.46 ± 0.06^Ab^	9.27 ± 0.07^Bb^	38.38 ± 1.04^Ab^	17.72 ± 0.81^Bb^
90	9.23 ± 0.09^Ac^	8.97 ± 0.06^Bc^	19.04 ± 1.75^Ac^	13.85 ± 0.68^Bc^
120	8.97 ± 0.09^Ad^	8.36 ± 0.13^Bd^	8.83 ± 0.83^Ad^	4.74 ± 0.93^Bd^
150	8.69 ± 0.10^Ae^	7.72 ± 0.22^Be^	3.61 ± 0.47^Ae^	1.38 ± 0.50^Be^

Different capital letters indicate significant differences between different digestive sites at the same digestion time (*p* < 0.01); different lowercase letters indicated that there was a significant difference in digestion time between the same digestive site (*p* < 0.05).

### 3.5 Effects of different digestion times on the contents of active substances in fermented honeysuckle liquid

**Figure 5:**
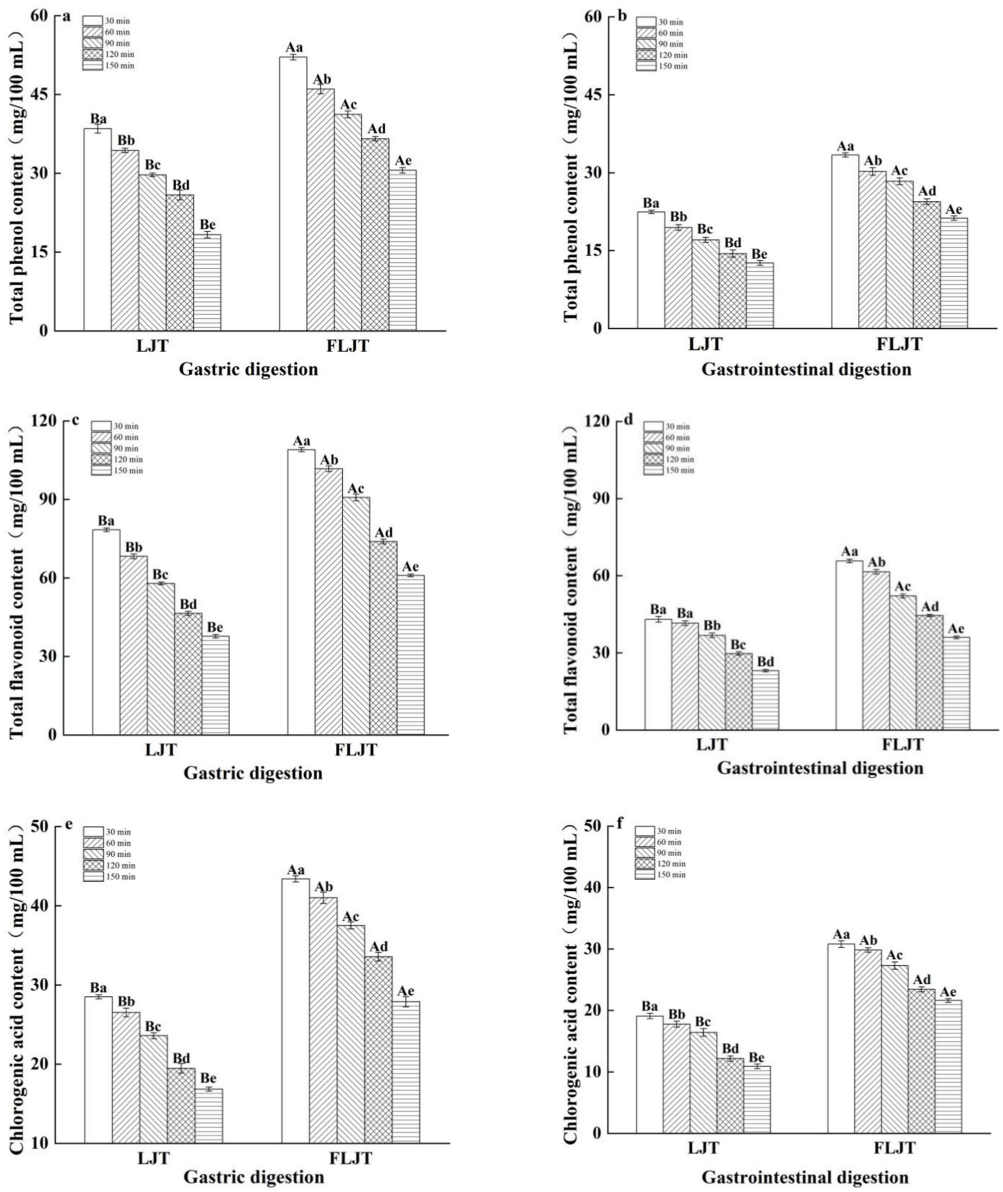
Effects of different digestion time of DIVRS model on the contents of bio-active components of fermented honeysuckle extract: **(a,c,e)** gastric digestion phase; **(b,d,f)** gastrointestinal digestion phase. Different capital letters in the broken line indicate significant differences between different digestive sites at the same digestion time (*p* < 0.01); different lowercase letters in the broken line indicated that there was a significant difference in digestion time between the same digestive site (*p* < 0.05).

The contents of total phenols, total flavonoids and chlorogenic acid in the fermented honeysuckle liquid and the unfermented honeysuckle liquid gradually decreased with increasing digestion time during digestion in the DIVRS model (*p* < 0.05) (Figure 5). The total polyphenol contents of LJT and FLJT were 38.48 mg/100 mL and 52.09 mg/100 mL, the total flavonoids contents were 78.42 mg/100 mL and 108.98 mg/100 mL, and the chlorogenic acid contents were 28.50 mg/100 mL and 43.39 mg/100 mL, respectively, after gastric digestion for 30 min. After digestion for 150 min, it decreased to 18.29 mg/100 mL and 30.52 mg/100 mL, 37.65 mg/100 mL and 60.99 mg/100 mL, respectively. 16.83 mg/100 mL and 27.87 mg/100 mL. The total polyphenol content of LJT and FLJT after 30 min of gastrointestinal digestion was 22.43 mg/100 mL and 33.42 mg/100 mL, respectively. The total flavonoid content was 43.04 mg/100 mL and 65.68 mg/100 mL, and the chlorogenic acid content was 19.07 and 30.81, respectively, which decreased to 12.61 mg/100 mL and 21.23 mg/100 mL after digestion for 150 min, respectively. 23.07 mg/100 mL and 36.01 mg/100 mL, 10.91 mg/100 mL and 21.60 mg/100 mL possibly because of the continuous secretion of simulated gastric juice and simulated intestinal juice during digestion. A continuous increase in simulated gastric juice and simulated intestinal juice will lead to the gradual dilution of the concentrations of total phenols, total flavonoids and chlorogenic acid in honeysuckle juice. However, the contents of total phenols, total flavonoids and chlorogenic acid in fermented honeysuckle liquid were always greater than those in unfermented honeysuckle liquid during the whole digestion process, which may be due to the increase in these bioactive components in the fermented honeysuckle liquid resulting from the fermentation of lactic acid bacteria. These factors increased the content of active components in the fermented honeysuckle liquid compared to that in the unfermented honeysuckle liquid, even in complex digestion environments.

### 3.6 Effects of different digestion times on the antioxidant activity of the honeysuckle liquid

**Figure 6:**
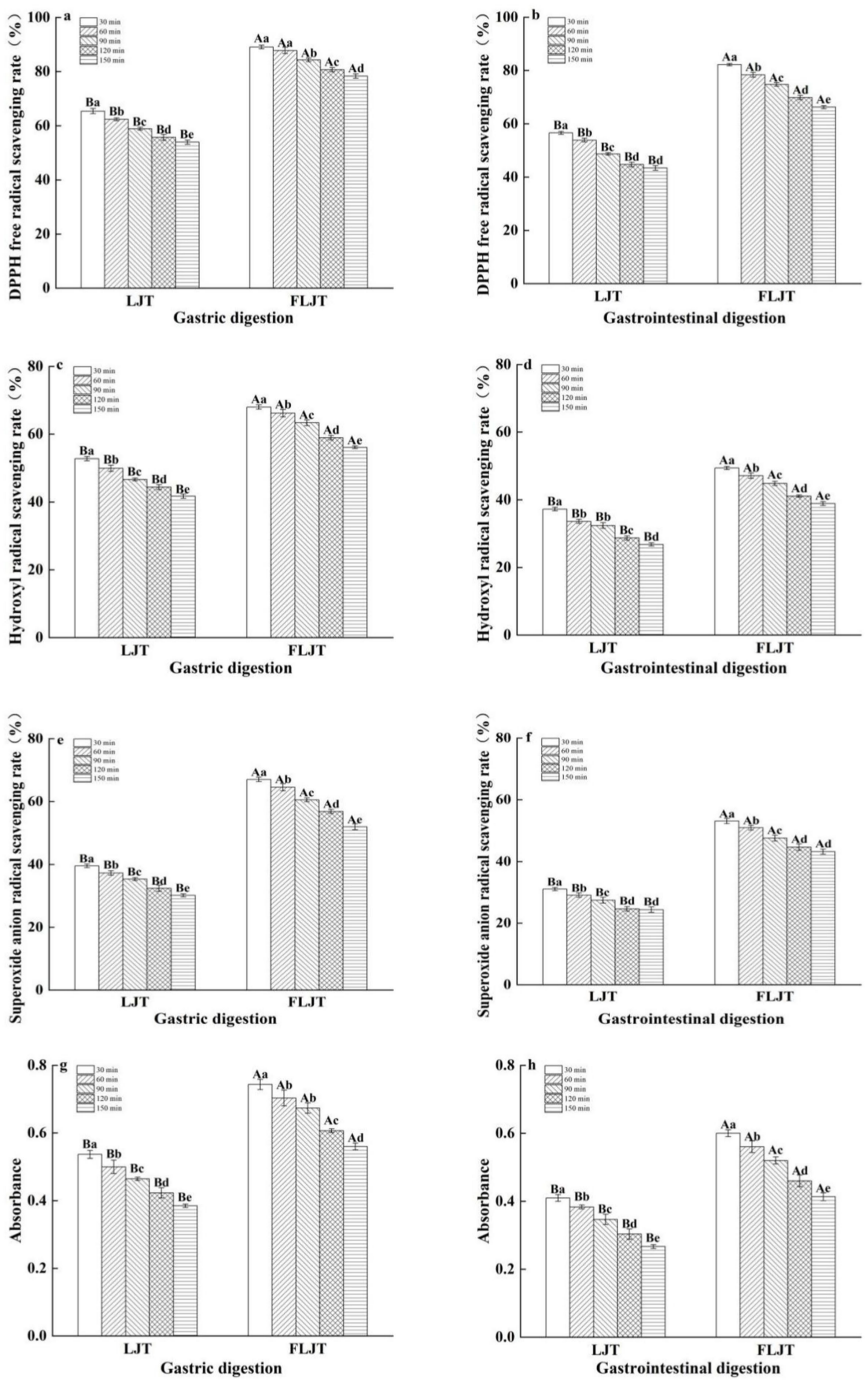
Effects of different digestion time of DIVRS model on the antioxidant capacity of fermented honeysuckle extract: **(a,c,e,g)** gastric digestion phase; **(b,d,f,h)** gastrointestinal digestion phase. Different capital letters in the broken line indicate significant differences between different digestive sites at the same digestion time (*p* < 0.01); different lowercase letters in the broken line indicated that there was a significant difference in digestion time between the same digestive site (*p* < 0.05).

The changes in the antioxidant capacity of fermented honeysuckle liquid and unfermented honeysuckle liquid in the DIVRS digestion model are shown in Figure 6. The scavenging ability and reducing ability of DPPH, hydroxyl and superoxide anion radicals were measured to determine the changes in the antioxidant capacity of the honeysuckle solution during digestion. The antioxidant activity of both the fermented and unfermented honeysuckle liquids decreased gradually with increasing digestion time, which was consistent with the trend of the change in total phenol, flavonoid and chlorogenic acid contents during digestion, which indicated that the bioactive components of the honeysuckle liquid clearly affected the antioxidant activity during simulated gastrointestinal digestion. The scavenging ability and reducing ability of DPPH radicals, hydroxyl radicals and superoxide anion radicals in the fermented honeysuckle liquid decreased by 27.77, 45.04, 37.72, and 46.55%, respectively, after digestion for 150 min. The scavenging ability of DPPH radicals, hydroxyl radicals and superoxide anion radicals and the total reducing ability of the unfermented honeysuckle liquid decreased by 36.34, 52.47, 44.31, and 52.94%, respectively.

### 3.7 Effects of different digestion times on *α*-glucosidase inhibition by fermented honeysuckle liquid

**Figure 7:**
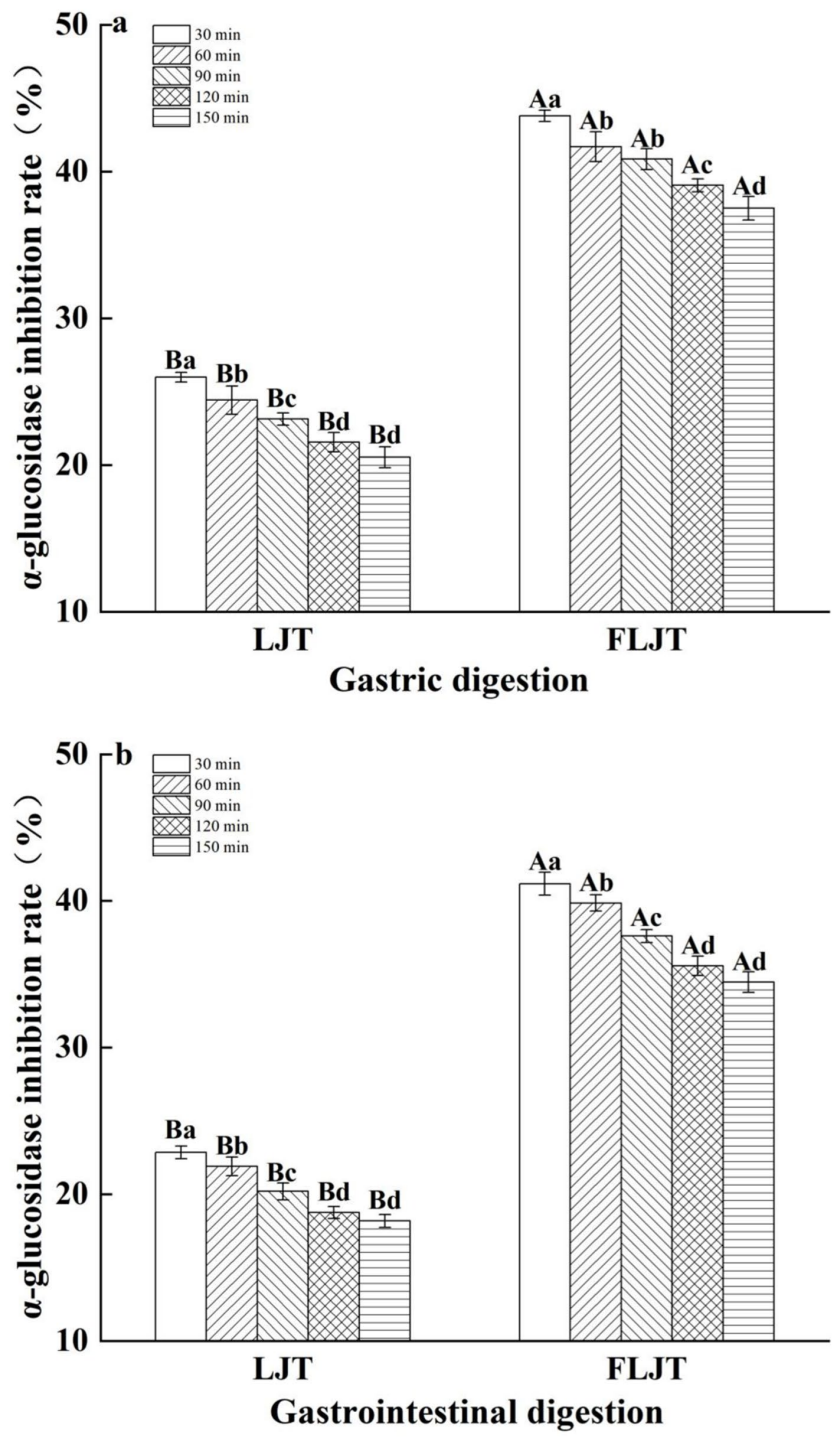
Effects of different digestion time of DIVRS model on α-glucosidase inhibition of fermented honeysuckle extract: **(a)** gastric digestion phase; **(b)** gastrointestinal digestion phase. Different capital letters in the broken line indicate significant differences between different digestive sites at the same digestion time (*p* < 0.01); different lowercase letters in the broken line indicated that there was a significant difference in digestion time between the same digestive site (*p* < 0.05).

The effects of the fermented honeysuckle liquid and unfermented honeysuckle liquid on α-glucosidase inhibition during digestion are shown in Figure 7. The inhibitory effects of the fermented honeysuckle liquid and unfermented honeysuckle liquid on α-glucosidase exhibited different degrees of change during digestion. The inhibition rates of α-glucosidase by the fermented honeysuckle liquid and unfermented honeysuckle liquid were 34.46 and 18.17%, respectively, after digestion for 150 min. Compared with that in the unfermented honeysuckle liquid, the inhibition rate of α-glucosidase by the fermented honeysuckle liquid was increased by 47.27%, which indicated that the fermented honeysuckle liquid had a better ability to inhibit α-glucosidase after digestion.

### 3.8 Effect of gastrointestinal digestion on the bioavailability of active substances in honeysuckle liquid

**Figure 8:**
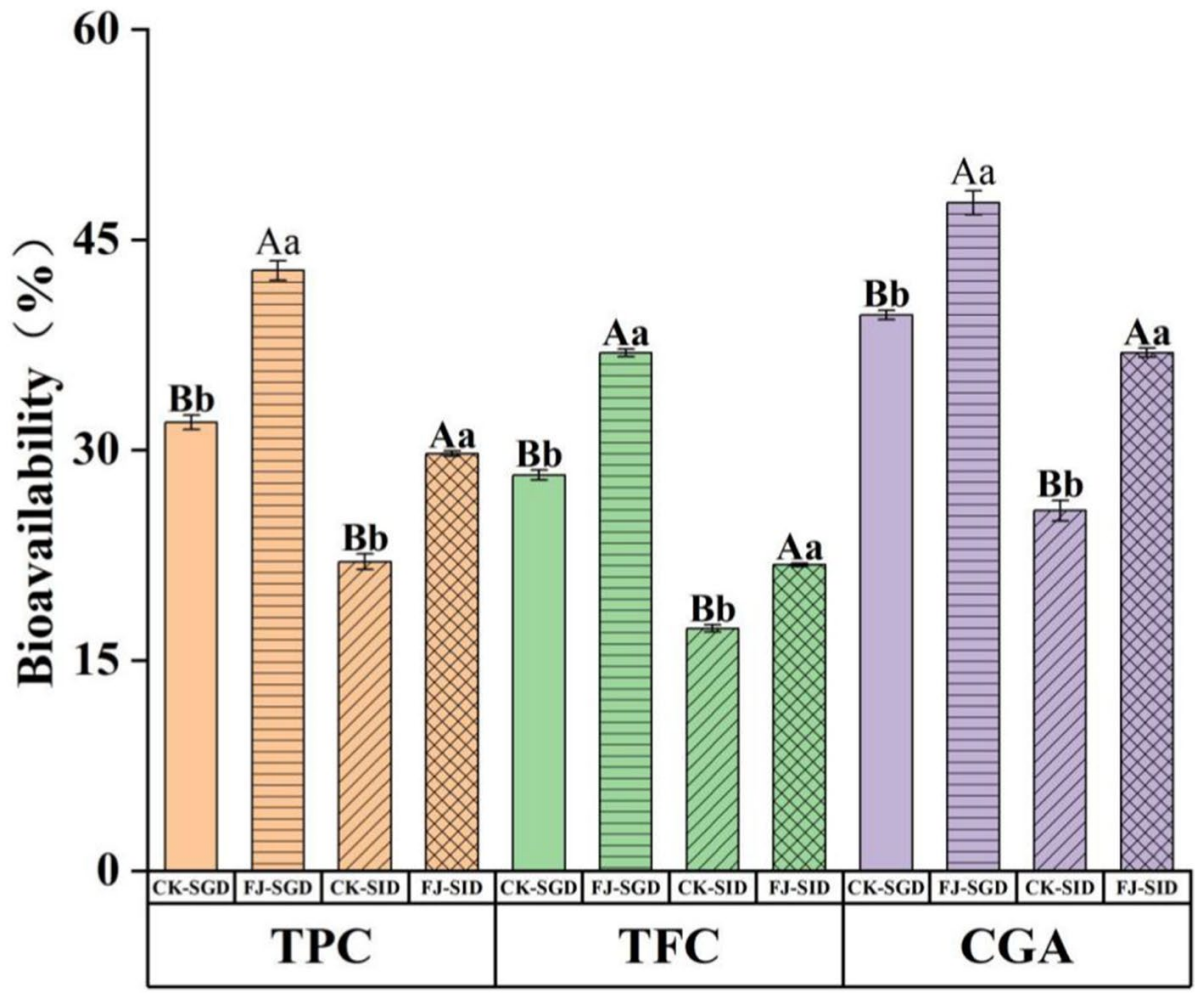
Effects of DIVRS digestion on the bio-availability of the bio-active components in fermented honeysuckle extract. TPC is total polyphenols content, TFC is total flavone content. CGA is chlorogenic acid content. Different capital letters indicate significant differences between different digestive sites at the same digestion time (*p* < 0.01); different lowercase letters indicated that there was a significant difference in digestion time between the same digestive site (*p* < 0.05).

In this study, the contents of total phenols, total flavonoids and chlorogenic acid in the fermented honeysuckle liquid and unfermented honeysuckle liquid were determined during digestion, and the bioavailabilities of these compounds were calculated. The results are shown in Figure 8. After *in vitro* digestion, the bioavailabilities of total phenols, total flavonoids and chlorogenic acid were 29.72, 21.80, and 36.93%, respectively. At the same stage, the bioavailabilities of total phenols, total flavonoids and chlorogenic acid in the unfermented honeysuckle liquid were 22.03, 17.28, and 25.67%, respectively. The increase in the bioavailability of active substances in the honeysuckle liquid after fermentation may be due to the decomposition of insoluble or combined compounds during fermentation, which increased the content and types of free phenols. At the same time, the chemical structure of the compounds changed, which made them easier to digest and absorb, thus increasing their solubility and bioavailability during digestion; otherwise, it may be due to lactic acid bacteria fermentation producing new metabolites, which further promoted the absorption and utilization of phenols and flavonoids.

### 3.9 Changes in the quality of fermented honeysuckle beverage during storage

**Figure 9:**
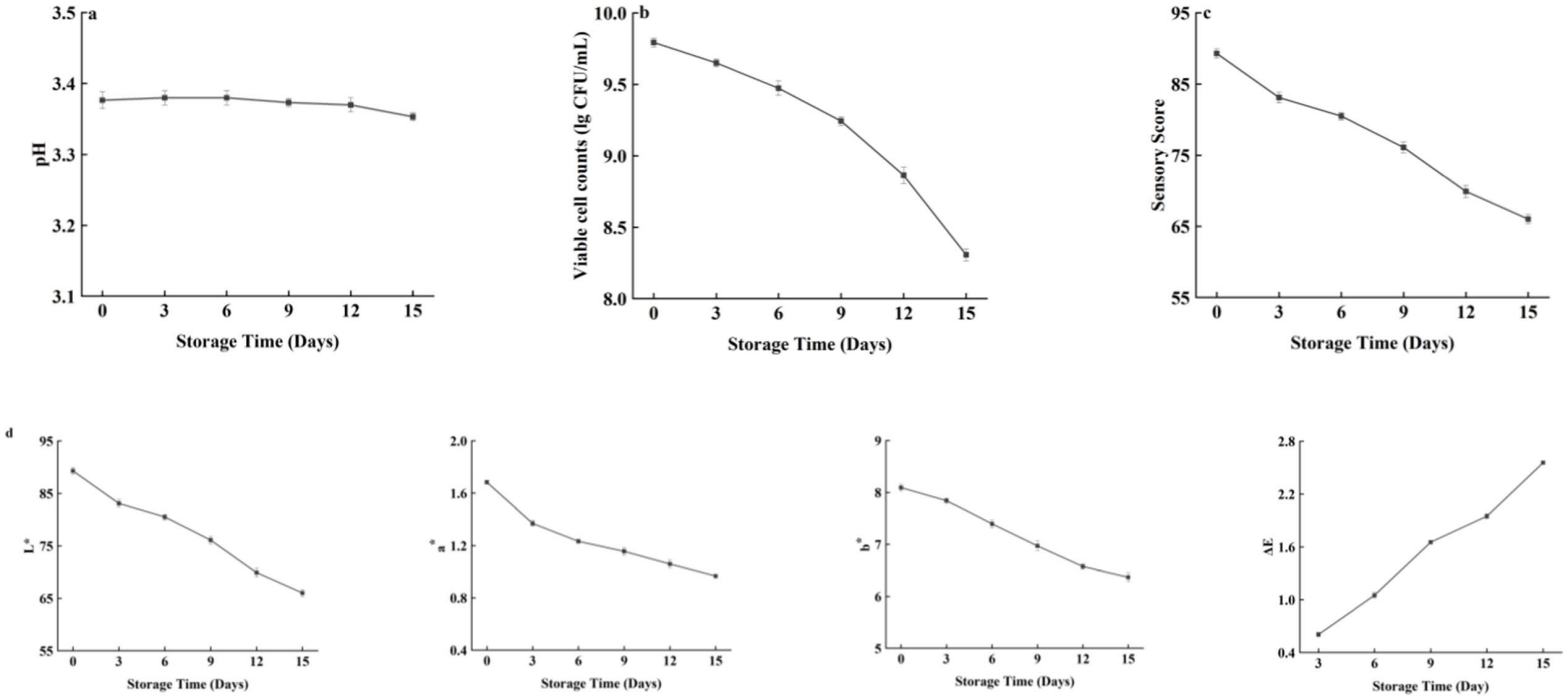
Effect of different storage time on the pH/viable bacteria/sensory score/color characteristics of fermented honeysuckle beverage.

pH is one of the important factors affecting the quality of beverages, and the change in pH is an important index for measuring the quality of beverages during storage; thus, the change in beverage quality can be judged by studying the change in pH during storage. The pH changes in fermented honeysuckle beverages during storage are shown in Figure 9a. The pH of the fermented honeysuckle beverage remained relatively stable during storage at 4°C, with no significant change (*p* > 0.05).

The changes in the number of viable bacteria during storage of the fermented honeysuckle beverages are shown in Figure 9b. As the storage time increased, the number of viable bacteria in the fermented honeysuckle beverage decreased gradually (*p* < 0.05). The number of viable bacteria in the fermented honeysuckle beverage was 9.79 lg CFU/mL at the beginning and 8.31 lg CFU/mL at 4°C after 15 days, which was a decrease of one order of magnitude. In addition, acidic environments may also inhibit the growth and metabolism of lactic acid bacteria. After 15 days of storage at 4°C, the viable bacteria count of the fermented honeysuckle beverage remained above 10^6^ CFU/mL, which met the standard requirements of viable bacteria count in live lactic acid bacteria beverages.

The changes in the sensory scores of the fermented honeysuckle beverages during storage are shown in Figure 9c. As the storage time of the fermented honeysuckle beverages increased, the sensory score of fermented honeysuckle beverages gradually decreased (*p* < 0.05). At the initial stage of storage, the fermented honeysuckle beverage had a moderate sweet and sour taste, a fresh taste, a bright soup color, no impurities, a uniform color and no stratification. After 15 days of storage at 4°C, the fermented honeysuckle beverage experienced some delamination, but it had a good taste, no obvious color change and a high sensory acceptability, and the precipitate disappeared after shaking well. Low temperature can maintain the color, taste and taste of beverages and delay the decrease in the sensory quality of beverages.

The changes in the color characteristics of the fermented honeysuckle beverages during storage are shown in Figure 9d. With increasing storage time, the L*, a*, and b* values of the fermented honeysuckle beverage gradually decreased, whereas ΔE gradually increased (*p* < 0.05), which indicated that the fermented honeysuckle beverage tended to darken during storage, which may be related to phenolic oxidation and other nonenzymatic browning reactions. However, the ΔE of the fermented honeysuckle beverage was less than 3.5 after being stored at 4°C for 15 days, which indicated that the overall color of the fermented honeysuckle beverage changed little during storage.

## 4 Discussion

In this study, *L. acidophilus* zrx02 was used as the fermentation strain, and the basic fermentation conditions of the honeysuckle liquid fermented by lactic acid bacteria were a fermentation temperature of 35°C, fermentation time of 19 h and inoculum amount of 3%. The pH of the fermented honeysuckle beverage was relatively stable during storage at 4°C, and the number of viable bacteria, sensory score and color decreased. The pH environment is beneficial for reducing the degradation of phenols and flavonoids (25). Whether probiotics can play a probiotic role *in vivo* is related to their survival rate in the host gastrointestinal tract. Only when probiotics can colonize the gastrointestinal tract environment and survive effectively can they have health benefits. Related studies have shown that probiotics can promote human health only when the amount of probiotics in the intestinal tract reaches more than 6 lg (CFU/mL) ~ 7 lg (CFU/mL) (26) Therefore, evaluating the changes in the number of viable bacteria and the survival rate of lactic acid bacteria during simulated gastrointestinal digestion of fermented honeysuckle liquid is highly important. The results indicated that a considerable number of lactic acid bacteria can tolerate the gastrointestinal environment better, maintain good survival ability and play a probiotic role in the gastrointestinal tract.

The contents of total phenols, total flavonoids and chlorogenic acid in fermented honeysuckle liquid and unfermented honeysuckle liquid gradually decreased with increasing digestion time during digestion in the DIVRS model. The microbial deglycation effect of glycosylated phenolic compounds, followed by the release of soluble conjugated phenolic compounds or insoluble bound phenolic compounds from plant cell walls, increased (27); otherwise, polyphenols and flavonoids with complex structures are also decomposed into anthocyanins, chlorogenic acid, flavonoids and their derivatives by enzymes during fermentation. Moreover, the decomposition of various digestive enzymes during digestion promotes further transformation and release of bioactive components in honeysuckle beverages. In addition, the pH of the fermented honeysuckle solution during gastrointestinal digestion is always in a more stable state than that of the unfermented honeysuckle solution. A stable pH is beneficial for reducing the autoxidation and degradation rates of active ingredients (28). In the process of digestion, the content of active ingredients in the intestinal tract is always lower than that in the stomach (*p* < 0.05), as phenols are sensitive to the digestive environment of the intestinal tract under intestinal digestion conditions, which leads to a decrease in its stability, and the degradation amount exceeds the release amount, resulting in a decrease in the content in the intestinal tract (29). Studies have shown that phenols are more stable in acidic environments in the stomach and that the activity of pepsin is beneficial for the release of bound phenols from the food matrix (30). The increased dietary fiber and mineral ions from digestion may combine with phenols and flavonoids, resulting in a gradual decrease in total phenols, total flavonoids and chlorogenic acid during digestion (31). This finding was consistent with the results obtained by Li et al. (32) in the *in vitro* digestion of fermented kiwifruit.

There is a positive correlation between antioxidant activity and active substances such as chlorogenic acid, caffeic acid, luteolin, quercetin and so on, the phenolic substances are found in fermented honeysuckle beverage, and related studies have reported that the gastrointestinal digestion process has different effects on phenols and flavonoids and their monomeric components, especially in the simulated gastrointestinal digestion process *in vitro* (33). Although the antioxidant activity of the fermented honeysuckle solution decreased during digestion compared with that of the unfermented honeysuckle solution, the fermented honeysuckle solution had better tolerance to the gastrointestinal tract and always presented better free radical scavenging activity and reducing ability during digestion, which indicated that the fermented honeysuckle solution has great potential for improving antioxidant activity. However, there was no identification of individual phenolic substances in this study. In order to further study the changes of phenolic substances during the fermentation and digestion of honeysuckle beverage, HPLC-MS method could be employed to separate and quantify these substances.

Rodríguez-Solana et al. (34) reported that foods with *α*-glucosidase inhibition ability are ideal foods for treating postprandial hyperglycemia and that inhibiting α-glucosidase activity can alleviate oxidative stress caused by chronic hyperglycemia in diabetes, thus preventing or reversing diabetic complications. In this study, the gastrointestinal-digested honeysuckle liquid had good α-glucosidase inhibitory ability, especially the fermented honeysuckle liquid, which may also be due to the bioactive components in the fermented honeysuckle liquid. Inada et al. (35) reported that the bioavailability of total phenolic compounds in Jaboticaba peel and seeds was 47% after gastric digestion, whereas the bioavailability of total phenolic compounds decreased to 19% after intestinal digestion. Chait et al. (36) reported that the total flavonoid content in digested carob decreased significantly, with decreases of 25.6, 51.2, and 58.1% during oral, gastric and intestinal digestion, respectively. The reason for the decrease in chlorogenic acid during digestion may be that chlorogenic acid can be isomerized into neochlorogenic acid and cryptochlorogenic acid under digestive tract environmental conditions (37). Some studies have shown that isomers of cryptochlorogenic acid may alleviate inflammatory symptoms and inhibit oxidative stress (38). Therefore, the biotransformation of chlorogenic acid in the honeysuckle liquid into neochlorogenic acid and cryptochlorogenic acid in the digestive tract environment has potential benefits for improving human health.

The pH of the fermented honeysuckle beverage remained relatively stable during storage at 4°C, with no significant change (*p* > 0.05). This may be because lactic acid bacteria metabolize slowly in low-temperature environments and can maintain high activity in the early stage of storage. However, with increasing storage time of the fermented honeysuckle beverages, lactic acid bacteria gradually decline, and metabolites such as organic acids can no longer accumulate, so the pH remains relatively stable during storage, and in some cases, cells can be metabolically active but unable to multiply, VBNC (viable but non-cultivable cells) show low but detectable metabolic activity, maintain membrane integrity, and express genes at low levels but the formation of colony forming units (CFUs) on culture media is inhibited, the % of survival could be higher than that presented in the study. This finding is consistent with the research results of Lim et al. (39) on pH changes in fermented dragon fruit beverages during storage. The good quality of the fermented honeysuckle beverage was maintained when the beverage was stored at 4°C. The phenolic substances in the honeysuckle beverage may destroy the structure of the cell membrane, and the growth and survival ability of lactic acid bacteria are inhibited with increasing storage time of the fermented honeysuckle beverage, resulting in a gradual decrease in the number of viable bacteria (40).

This research on honeysuckle beverages fermented with lactic acid bacteria provides a reference basis for the development of honeysuckle products and specific functional probiotic products.

## 5 Conclusion

*Lactobacillus acidophilus* zrx02 was used to prepare the fermented honeysuckle beverage, and its digestive and functional characteristics were investigated. The fermented beverage had strong antioxidant and *α*-glucosidase inhibition ability, and its pH was relatively stable during storage at 4°C, and the number of viable bacteria and color decreased. The fermented beverage of honeysuckle flower has good functional characteristics and has a good market prospect.

## Data Availability

The original contributions presented in the study are included in the article/supplementary material, further inquiries can be directed to the corresponding author.
